# Non-Viral in Vitro Gene Delivery: It is Now Time to Set the Bar!

**DOI:** 10.3390/pharmaceutics12020183

**Published:** 2020-02-21

**Authors:** Nina Bono, Federica Ponti, Diego Mantovani, Gabriele Candiani

**Affiliations:** 1 GenT Lab, Department of Chemistry, Materials and Chemical Engineering “G. Natta”, Politecnico di Milano, 20131 Milan, Italy; nina.bono@polimi.it (N.B.); federica.ponti@polimi.it (F.P.); 2 Laboratory for Biomaterials and Bioengineering, Canada Research Chair I in Biomaterials and Bioengineering for the Innovation in Surgery, Department of Min-Met-Materials Engineering & Research Center of CHU de Quebec, Division of Regenerative Medicine, Laval University, Quebec City, QC G1V 0A6, Canada; diego.mantovani@gmn.ulaval.ca

**Keywords:** non-viral gene delivery, cationic polymers, PEI, polyplexes, in vitro transfection, physico-chemical characterization, variability, reproducibility, standardization

## Abstract

Transfection by means of non-viral gene delivery vectors is the cornerstone of modern gene delivery. Despite the resources poured into the development of ever more effective transfectants, improvement is still slow and limited. Of note, the performance of any gene delivery vector in vitro is strictly dependent on several experimental conditions specific to each laboratory. The lack of standard tests has thus largely contributed to the flood of inconsistent data underpinning the reproducibility crisis. A way researchers seek to address this issue is by gauging the effectiveness of newly synthesized gene delivery vectors with respect to benchmarks of seemingly well-known behavior. However, the performance of such reference molecules is also affected by the testing conditions. This survey points to non-standardized transfection settings and limited information on variables deemed relevant in this context as the major cause of such misalignments. This review provides a catalog of conditions optimized for the gold standard and internal reference, 25 kDa polyethyleneimine, that can be profitably replicated across studies for the sake of comparison. Overall, we wish to pave the way for the implementation of standardized protocols in order to make the evaluation of the effectiveness of transfectants as unbiased as possible.

## 1. Introduction

The delivery of nucleic acids (NAs) to mammalian cells (a process named transfection) has emerged as a new breakthrough in molecular medicine for the treatment at a genetic level of several diseases, including inherited disorders, some types of cancer, and certain viral infections [1,2,3,4], and in fundamental research and bionanotechnology for the investigation of basic cellular mechanisms or the production of specific proteins [5,6,7]. 

As a rule of thumb, gene delivery involves the deliberate modulation of gene expression patterns through the delivery of exogenous genetic material, such as (i) chimeric circular plasmid DNAs (pDNAs), which are hybrid plasmids with an expression cassette containing a specific gene of interest (such as a reporter gene encoding for an easily traceable protein, e.g., luciferase or a fluorescent protein), (ii) messenger RNA (mRNA), (iii) short regulatory RNAs such as short-interfering RNAs, micro RNAs, and short hairpin RNAs (siRNA, miRNA, and shRNA, respectively), and (iv) antisense oligonucleotides (ASOs) [1,8] into the target site of action (Table 1).

Although the delivery of naked NAs is considered the safest way to transfect cells, such a process is unfortunately ineffective. As a matter of fact, NAs cannot freely cross the cytoplasmic membrane because of the electrostatic repulsions occurring at physiological pH between the anionic NAs and the negatively charged plasma membrane. Yet, NAs suffer from limited extracellular stability. In fact, the genetic material is very prone to degradation by extracellular nucleases present in bodily fluids (in vivo) and in general extracellular milieu (in vivo and in vitro).

In order to address these issues, in the last decades, great efforts have been devoted to devising suitable approaches and tools to improve the delivery efficiency of NAs into target cells. Such strategies can be broadly classified into two main categories (Table 2), namely gene delivery methods and vectors [11,12].

Physical/mechanical methods, such as electroporation [13,14,15], sonoporation [16,17,18], magnetofection [19,20], optoporation [21,22], gene gun [23], and microinjection [24,25,26], attempt to force naked NAs into the cytosol or nucleus to achieve successful transfection [27,28]. Although conceptually simple yet powerful means for transfecting cells, such methods are however expensive and somehow inconvenient for most gene delivery applications [29]. In this survey, we deliberately avoid dealing with physical methods-mediated gene delivery.

The other popular way to deliver NAs into mammalian cells relies on the use of gene delivery carriers, also known as systems or vectors, which can be further categorized into viral and non-viral ones. An ideal gene delivery vector should be effective, specific, and safe [30,31,32,33].

As viruses are highly evolved biological systems that effectively gain access to host cells by nature, engineered viral vectors, that is, viruses in which the therapeutic gene cassette is in place of part of the viral genome, are so far the most widely used vehicles in gene therapy [34,35,36,37]. Despite these unique strengths, they are still plagued by inherent issues such as the limited size of NAs that can be packed and delivered, random recombination (i.e., oncogenic potential), cytotoxicity, and immunogenicity [38,39]. These concerns, together with the high costs related to large-scale production and quality control, have steered research towards non-viral carriers [40,41].

Since the inception of inorganic matter for plasmid delivery in the 1970s [49], the last decades have thus witnessed a surge of interest in non-viral systems [1,50,51,52]. Because they are relatively safe, display easily tunable physico-chemical properties, can be produced in large quantities with high reproducibility and affordable costs, and show unlimited ferrying capacity [53,54,55], non-viral vectors are nowadays at the forefront of gene delivery [1]. The two major approaches to non-viral gene delivery involve the combination of NAs with transfection molecules, that is, cationic lipids (CLs; e.g., DOTAP, DOTMA) [56,57,58,59,60,61] in different forms, and cationic polymers (CPs; e.g., poly-*L*-lysines, polyethyleneimines (PEIs), poly(amidoamine)s, chitosans) [62,63,64,65,66,67]. The beauty of this approach lies in its simplicity, yet in its effectiveness. Indeed, such positively charged (poly)electrolytes self-assemble with the anionic NAs to give rise to particle-like complexes (lipoplexes and polyplexes when complexation involves CLs and CPs, respectively), having sizes ranging from tens of nm to few µm, which are able to sneak through the cell membrane and allow NAs internalization into cells [12].

**Figure 1:**
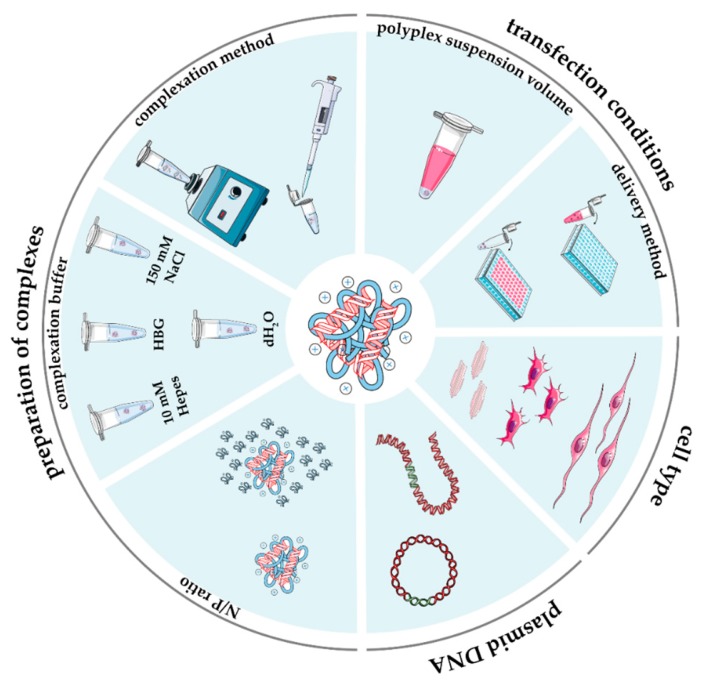
Main experimental parameters influencing the in vitro performance of gene delivery vectors.

With the use of contemporary tools and techniques, it has become possible to somehow tune the performance of non-viral vectors for gene delivery [68]. Nevertheless, despite a large variety of reagents that has been developed worldwide with some success (for comprehensive reviews, refer to [11,69,70,71]), the design of more and more effective gene delivery vectors is still painfully slow. Whatever the strategy used, success largely depends first on gathering reliable data from the literature. As for other biomedical subjects [72,73,74,75,76], lab-to-lab variability in routine transfection protocols in vitro (as depicted in Figure 1), that is, the lack of standard test procedures, has largely contributed to the huge number of inconsistent findings underpinning the reproducibility crisis. Indeed, scientists are flooded with data that are hard to digest, compare, and integrate into a clear view of how to design a transfectant with superior behavior.

Herein, we seek to highlight the issues involved and suggest ways in which this process can be expedited and improved. A key question is how robust the approaches are to screening and implementing new gene delivery vectors, and what measures could be taken into account to shorten the long and protracted development of new transfectants. This review seeks to provide the readers with a precise and clear catalog of issues that, to different extents, may affect the effectiveness of non-viral gene delivery vectors. A thorough survey of literature data and some genuine findings disclosed herein, taking as an example the complexes prepared using model molecules polyethyleneimine (PEI) and pDNA (for the exhaustive description of the experimental setups, please refer to the supplementary materials), aim to fill the multiple gaps in bibliographic information and shed light on the profound impact of common experimental parameters—too often neglected—on the in vitro transfection behavior of gene delivery vehicles. 

## 2. Non-Viral Gene Delivery Using Plasmid DNA and Cationic Polymers

### 2.1. Plasmid DNA

Whether considering the sole use of DNA, some variations in the transfection efficiency may rely on differences in the topology of the DNA being transferred. Previous studies from the Uludağ’s group have shed light on the greater transgene expression given by circular pDNA as compared to its linearized counterpart [77]. Similar findings have been reported for CLs as well [78]. Yet, other DNA topologies (e.g., supercoiled, open-circular, and linear) have been found to affect the expression of the transferred NAs [79]. Of note, the supercoiled pDNA (sc-pDNA) is widely recognized as the least susceptible to intracellular degradation and, as such, it is considered the most physiologically active pDNA to be used to transfect mammalian cells [79]. The mechanism underlying the influence of the DNA topology on the ultimate transfection efficiency is, however, still unknown. Nevertheless, it was found that sc-pDNA displays a very tiny hydrodynamic size that, in turn, may be responsible for its great intracellular mobility through entangled and cross-linked composite networks of actin and microtubules [78,79,80,81,82,83].

Generally speaking, pDNAs used for in vitro transfection studies vary not only in the specific reporter gene sequence they were endowed with (e.g., firefly luciferase, green fluorescence protein (GFP)), but also in other basic elements. An illustrative example of this issue relies on multicistronic constructs (i.e., pDNAs that express multiple genes at once, leading to the production of two or more separate proteins from the same mRNA) [84]. Alongside this, a specific promoter must be carefully selected in order to drive the constitutive overexpression of the transgene(s) in the specific host. Commercially sourced promoters, namely, human cytomegalovirus (CMV), simian vacuolating virus 40 (SV40), and elongation factor (EF)-1 [85,86,87], have shown some cell type-dependent specificity. Instead, it was shown that the CMV promoter invariably induces the highest transgene expression in any of the cell lines tested [88], and in differentiated cells, but was unable to do so in pluripotent stem cells [89].

Altogether, these findings allow us to pinpoint the features of a given gene construct that may affect the transfection outcomes in vitro. In this context, the most effective pDNA to be used for gene delivery purposes is in the form of sc-pDNA, which is typically obtained through its resuspension in 0.1× TE buffer (10 mM Tris-HCl, pH 8.0, and 1 mM disodium ethylenediaminetetraacetic acid (EDTA)) [90]. For a matter of consistency, reproducibility, and in order to ease the comparison of results, the exact name and/or code of the pDNA used for transfection have to be disclosed. As such, this implies that all the information about the size, promoter, and transgene expressed are to be made public. Furthermore and whenever possible, any noticeable information about pDNA (super)coiling, or at least information about the resuspension buffer used, must be very detailed.

### 2.2. Cationic Polymers (CPs)

**Figure 2:**
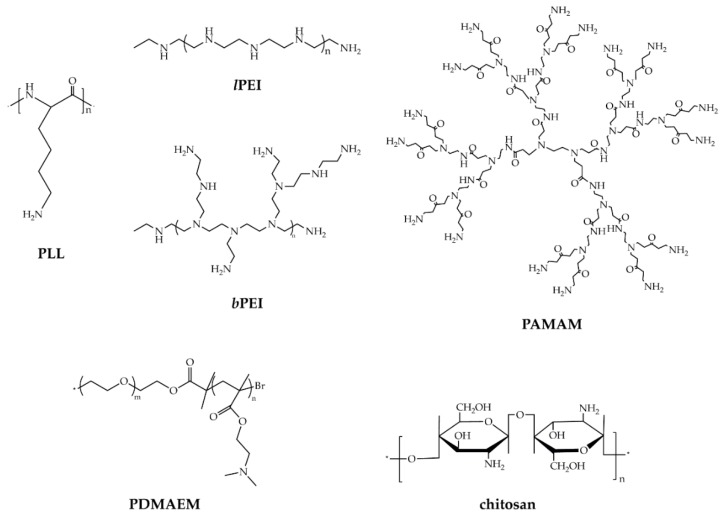
Chemical structures of commonly used cationic polymers for gene delivery purposes.

In the last decades, huge efforts have been devoted to engineering and identifying the ideal vector able to overcome the major physiological bottlenecks of gene delivery, namely cellular internalization, endosomal escape, pDNA translocation from the cytosol to the nucleus, and NAs release [68]. CPs are amongst the most utilized non-viral vectors for gene transfer (for comprehensive reviews on polymeric carriers, see [9,69,91,92]), which consist of multiple, positively charged residues, such as primary, secondary, tertiary amines, as well as amidines, guanidino, and triazino groups, which act as DNA-binding moieties during polyplexes formation. Besides, they can display different chemistries and architectures [93]. CPs include commercially sourced materials (Figure 2), such as poly-L-lysines (PLLs) [94,95], poly(ethyleneimines) (PEIs) [96,97], poly(amidoamines) (PAMAMs) [64,98,99,100,101], poly[2-(dimethyamino)ethyl methacrylates] (PDMAEMs) [102], and chitosans (CSs) [103,104,105,106], used as received or reacted and functionalized [63,107,108,109,110], and those purposely synthesized [111]. 

Of note, the efficacy of a transfectant strongly depends on some chemical and geometrical features. PEI is one of the most utilized non-viral polymeric vectors for gene delivery applications [69,112] due to its extremely high cation charge density. Depending on the way that the repeating ethylenimine units link together, that is, the way of polymerization, it occurs as linear (*l*PEI) or branched (*b*PEI) isomers (Figure 2) [111]. Other features, such as the degree of polymerization (molecular weight (M_W_), branching degree in the case of *b*PEI only, polydispersity) and the buffering capacity, which is strictly related to the cationic charge density, have an impact on the vector performances [112]. For instance, while *l*PEI and *b*PEI of equal M_W_ show similar transfection efficiencies [113], there is no general consensus as to which is the most effective M_W_ of PEI in transfection. Some authors have indeed reported that the transfection efficiency of *b*PEI in vitro increased with increasing the M_W_ of the CP (in the range from ≈1.8 to ≈70 kDa) [62,114], while others have highlighted that the lower the M_W_ of the branched polymer (≈12 kDa), the greater the effectiveness [115]. Further, we and some others have pointed out the different pDNA complexation ability of *l*PEI and *b*PEI and have found that, irrespective of the M_W_ of the CP taken into account, lPEIs complex best at N/P 5, while bPEIs at N/P 3 [57,62,64,116,117]. This different behavior may be ascribed to the different structures of the PEI isomers: of note, *l*PEI possesses only secondary amines, while bPEI has primary, secondary, and tertiary amines depending on the polymerization degree [118]. These account for the buffering capacity of the *b*PEI over a wide range of pH and, in turn, for the “proton-sponge effect” underpinning the escape of polyplexes from the endo/lysosomes. 

Overall, the good gene delivery performances of PEIs, together with moderate cytotoxicity they exert, disclose this class of CPs as gold standard transfectants. As such, they have been largely used as benchmark transfectants in most of the in vitro studies [57,112]. As a rule of thumb, according to ISO norms, reference materials are those whose properties are sufficiently homogeneous and well established to be used for assigning values to other materials [119,120], which in practice means to other transfectants being developed. This notion implies that a reference material has to be used under very defined and controlled conditions so that its activity is kept as constant as possible. In reality, there remain substantial inter-laboratory variability issues related to the effectiveness of PEI-based transfections that may depend on the gene delivery protocols adopted, the use of different raw materials (e.g., the type and dose of pDNA used, specific transfectant features), various pDNA/transfectant complexation conditions (e.g., N/P; complexation buffer; complexation temperature and time), specific complex doses administered to cells and cell culture conditions, different read-out systems, among others (Figure 1). All of this makes it dramatically difficult to fairly compare results between laboratories.

### 2.3. Preparation of Complexes

Despite the increasingly sophisticated analytical techniques available to evaluate the physico-chemical properties and biological behavior of gene delivery assemblies, real progress in the field of non-viral gene delivery is rather limited. However, this issue is symptomatic of far broader challenges in biomedical research that cannot be addressed by simply identifying suitable methods or techniques to carry out a given analysis [121]. Rather, we have brought some features into prominence, i.e., the DNA complexation ability of a CP, the size and surface charge of complexes, the milieu in which complexation occurs, that play a causative role in the transfection effectiveness of the resulting complexes. These will, therefore, be dealt with separately herein below.

#### 2.3.1. Cationic Polymer-to-Plasmid DNA Ratio

It is a matter of fact that the performances of polyplexes are largely dependent on the (mole or mass) ratio between the transfectant used for complexation and the NAs to be packed and delivered. Complexes are prepared by mixing a CP with pDNA while taking into account that the cationizing groups (typically amines) of the transfectant are the only moieties able to bind electrostatically to the anionic phosphates of the NA. In the most common and simplest case, such charge ratio is basically the ratio between the basic nitrogen (N) moles of the transfectant and the phosphate (P) moles borne by a given pDNA quantity and is referred to as the N/P ratio [122]. To put this definition into practice, we take for example the PEI molecule. Every basic amino group of the CP has to be regarded as potentially responsible for DNA binding, that is, one N per repeat unit of PEI ( -NHCH_2_CH_2_-, M_W_ = 43 Da) [62,123]. For a pDNA (i.e., a dsDNA), instead, one can calculate the phosphate (P) moles according to the Equations (1) and (2):

(1) P moles=dsDNA moles ×dsDNA length (bp)×2

where

(2) $$dsDNA\;moles\;=\;\frac{dsDNA\;mass\;\left(g\right)}{dsDNA\;length\;\left(bp\right)\times MW_{bp}}$$

As a general rule, there are ≈3 nmol of P every µg of pDNA.

**Figure 3:**
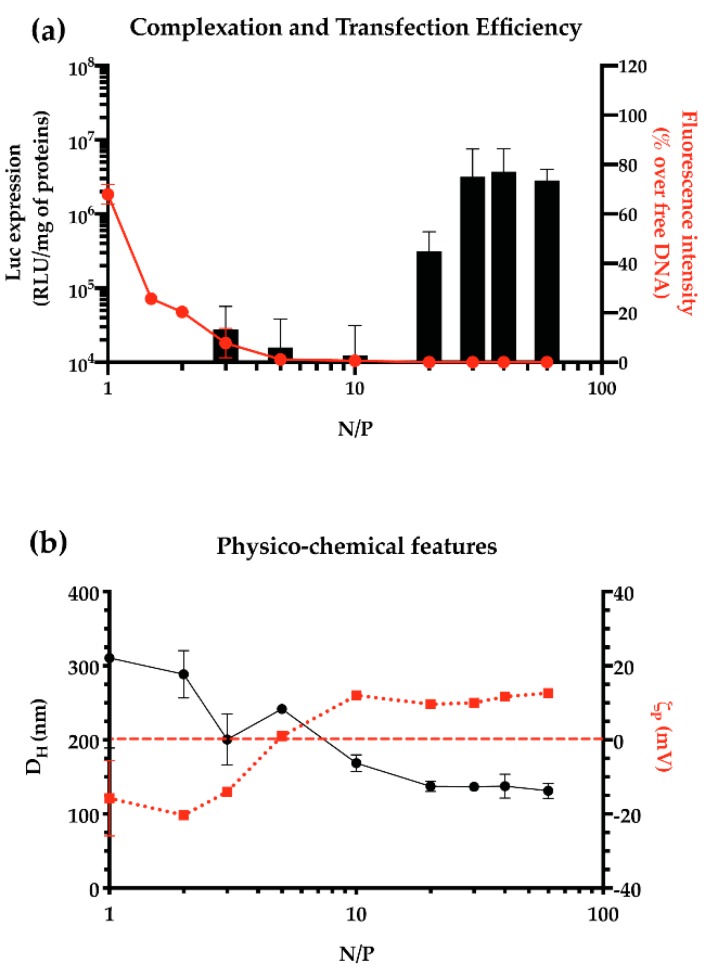
DNA complexation ability, transfection efficiency, and physico-chemical characteristics of pDNA/*l*PEI complexes prepared in 10 mM Hepes at different N/Ps. (**a**) Transfection efficiency (black bars) in L929 cells of pDNA/*l*PEI complexes prepared at different N/Ps. Results are expressed as luminescence signal (RLU) normalized to the total protein content in each cell lysate, and the DNA complexation ability of *l*PEI (red dots, solid line), evaluated by monitoring the fluorochrome exclusion from complexes as a function of the N/P. (**b**) Mean hydrodynamic diameter (D_H_, black dots solid line) and overall surface charge (ζ_P_, red squares and dotted line) of pDNA/*l*PEI complexes at different N/Ps, as measured by dynamic light scattering (DLS) and electrophoretic light scattering (ELS), respectively. Results are expressed as mean ± SD (*n* ≥ 3).

When dealing with the in vitro screening of transfection agents, the minimum ratio at which the pDNA is fully complexed into particles must be pinpointed, so that no naked and ineffective NA is delivered to cells. It is worth noting that this conditio seldom gives rise to the most effective particles in transfection experiments. For instance, if considering the model *l*PEI, the transfection efficiency of polyplexes increases along with the N/P (Figure 3a). We can speculate that the presence of single free PEI molecules in solution (at N/P 10, ≈50% of PEI is in the form of free CPs) contributes to the stability of the particles, in terms of ultimate size (usually expressed as hydrodynamic diameter, D_H_) and surface charge (usually expressed as zeta potential, ζ_P_) (Figure 3b), and destabilizes the cellular membrane, promoting the internalization of polyplexes and improving the transfection efficiency [117].

Thus, when evaluating the performance of a gene delivery vector, one should first find out the minimum N/P for effective complexation of a given amount of DNA, and next, the optimal N/P that allows for the greatest transfection and the lowest cytotoxicity in vitro.

#### 2.3.2. Polymer Solubilization and Complexation Buffer

Because non-viral gene delivery particles are formed by electrostatic interactions [111,124], they are sensitive to the composition of the medium (i.e., the saline composition, the ionic strength, and the pH) in which the complexation occurs. The most widely used buffers for complexation are 10 mM Hepes [62,96], whether supplemented or not with 5% (*w/v*) glucose (hereafter referred to as HBG buffer) [125], 150 mM NaCl [126,127], and deionized water (dH_2_O or MilliQ) [128]. 

**Figure 4:**
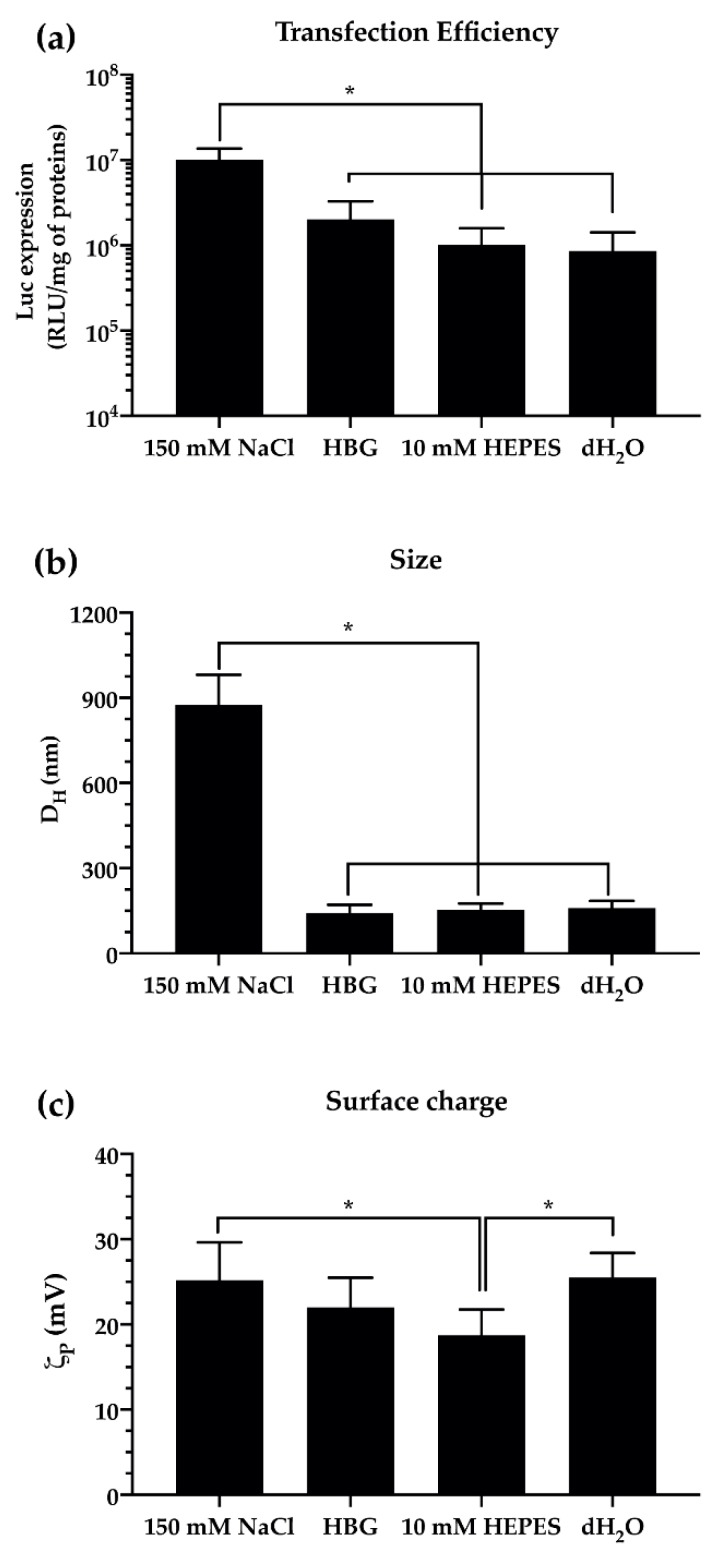
Effect of complexation buffer on the transfection efficiency and physico-chemical features of pDNA/*l*PEI polyplexes prepared at N/P 30 in L929 cells. (**a**) Transfection efficiency of pDNA/*l*PEI complexes prepared in different buffers. Complexes were prepared by adding 160 ng/cm^2^ of pGL3 to the *l*PEI solution. (**b**) Hydrodynamic diameter (D_H_) and (**c**) overall surface charge (ζ_P_) of pDNA/*l*PEI complexes prepared by adding 1 μg of pDNA to the *l*PEI solution, then complexes were diluted in different buffers. Measurements were carried out by means of a dynamic light scattering (DLS; for D_H_ measurements) and electrophoretic light scarring (ELS; for ζ_P_ measurements) apparatus. Results are expressed as mean ± SD (n ≥ 3) (* *p* < 0.05).

The transfection efficiency profiles (Figure 4a) observed in vitro when using pDNA/*l*PEI complexes prepared in different buffers reflect their different physico-chemical features in a size- and surface charge-dependent manner (Figure 4b,c, respectively). Literature data and our own findings agree that, in the presence of physiological salt concentrations (e.g., 150 mM NaCl), 25 kDa *l*PEI forms large polyplexes of ≈1 µm in size. Instead, rather smaller complexes of ≈200 nm are obtained in dH_2_O and a low-salt buffer (10 mM Hepes). On the other hand, the addition of glucose to the latter (i.e., HBG) allows us to obtain smaller pDNA/*l*PEI assemblies with a D_H_ of ≈100 nm (Figure 4 and Table S1). It is worth noting that the greater the particles, the faster their settling and the higher the transfection efficiency in adherent cells. Hence, *l*PEI-based complexes prepared in 150 mM NaCl (i.e., those with the greatest hydrodynamic size (Figure 4b)) settle onto cell monolayers in a similar way that the calcium phosphate-mediated transfection does [129], resulting in an ultimate transfection efficiency ≈10-fold higher than the other conditions (Figure 4a). Likewise, polyplexes with higher surface charge (i.e., ζ_P_) are much more effective in transfecting adherent cells in vitro (see Figure 4a,c, and [130]) due to their favorable binding with the cell membrane.

Given the above, it is apparent how the physico-chemical features and the actual biological effectiveness of non-viral gene delivery assemblies in vitro can be fine-tuned as a function of the ionic strength and the overall salinity of the dispersing medium. Besides, as the ionic strength dramatically affects the amine protonation, which impacts the interactions between PEI molecules and NAs or cell membranes [131], the way PEIs are solubilized in aqueous solutions and buffered at physiological pH are very fundamental facets that deserve some attention. Such cues should all be given due consideration when preparing PEI-based complexes and should be explicitly stated in the manuscript. In addition, the physico-chemical features of complexes should also be systematically evaluated in the context of their use, such as in the biological medium where transfection assays will be carried, and the type of cells used (anchorage-dependent, adherent vs. suspension culture). Yet, the temporal evolution of the D_H_ when the polyplexes are diluted in the culture medium should also be taken into account. Indeed, because culture media are rich in serum proteins that adsorb onto the polyplex surface to give the so-called protein corona [96,122,132], the D_H_ of polyplexes evolves over time in such dispersants [133].

Different analytical technologies, each one with specific pros and cons [134], are currently used for the evaluation of the physico-chemical characteristics of gene delivery complexes, such as atomic force microscopy (AFM), scanning electron microscopy (SEM), transmission electron microscopy (TEM), microfluidic resistive pulse sensing (MRPS), and the most widely used dynamic light scattering (DLS) [135]. In order to make the characterization as thorough and comprehensive as possible, at least a couple of the above should be used.

#### 2.3.3. Complexation Method

**Figure 5:**
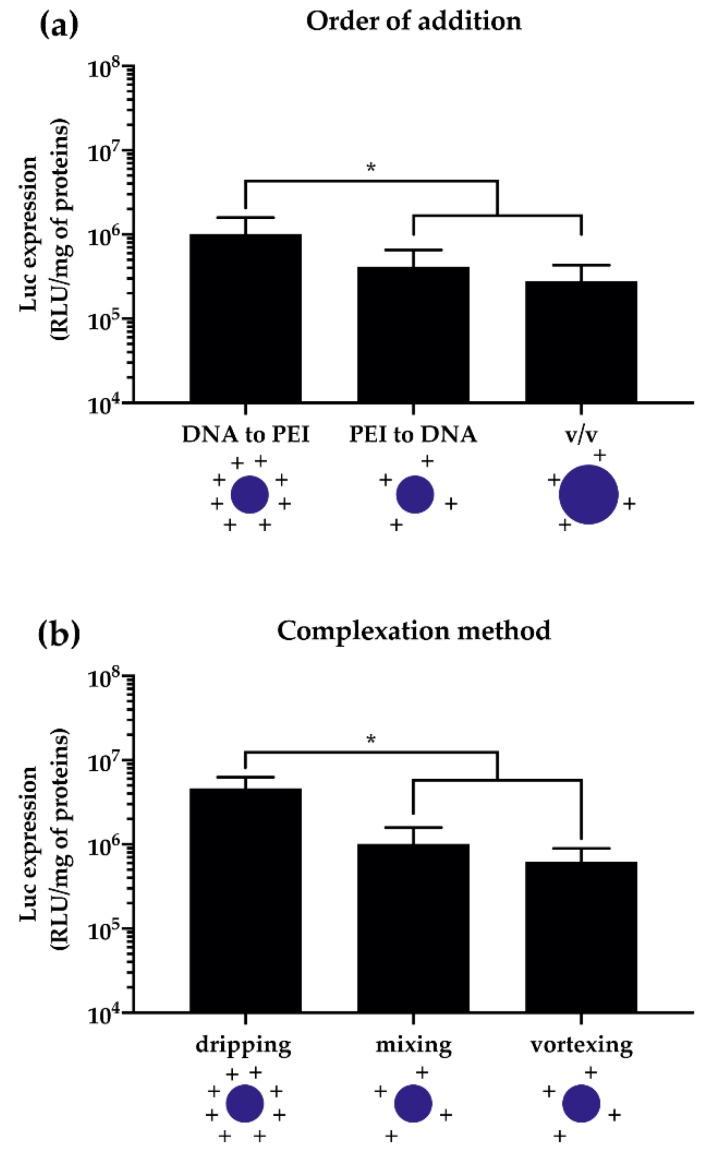
Effect of the complexation method on the transfection efficiency of pDNA/*l*PEI complexes at N/P 30 in L929 cells. (**a**) Transfection efficiency of pDNA/*l*PEI complexes as a function of the order of mixing and volumes of *l*PEI and pDNA solutions. Complexes were prepared by adding 160 ng/cm^2^ of pGL3 with the *l*PEI solution in 10 mM Hepes (DNA to PEI), or vice versa (PEI to DNA), then mixing the two solutions by rigorous pipetting, or by mixing equivolumes of DNA and PEI solutions (*v/v*). (**b**) Transfection efficiency of pDNA/*l*PEI complexes as a function of the complexation method. Complexes were prepared by adding 160 ng/cm^2^ of pGL3 to *l*PEI in 10 mM Hepes by single dripping, mixing (i.e., repeated and rigorous pipetting), and vortexing. Results are expressed as mean ± SD (*n* ≥ 3) (* *p* < 0.05).

One of the most underappreciated, if not neglected, issues is the dramatic effect that some minor changes in the way of blending the single components (i.e., the pDNA and the CP solutions) may have on the ultimate polyplex behavior in vitro. Indeed, sharply different complexes are generated by adding the pDNA solution to a large excess of transfectant solution (for instance at a ratio of ≈1:10 (*v/v*)) or vice versa, or when DNA solution is added to the PEI equivolume (*v/v*) (Figure 5a). Moreover, being the complexation an entropy-driven process [136], the way the pDNA is added to and mixed with the PEI solution, that is, (i) the addition of one solution to the other one and the stirring of the resulting mixture by means of repeated and rigorous pipetting, (ii) the single dripping of one solution into another and subsequent rest, and (iii) the vigorous vortex and stirring of the two solutions once blended together, does affect the transfection outcomes (Figure 5b).

Even though the reasons underpinning these disparate behaviors are still somewhat unclear, the dripping of the pDNA solution into the CP solution is the most straightforward way to produce very effective polyplexes.

Together, these findings entail that each and every material (i.e., the pDNA, the CP, and the compexation buffer) and the procedure used to prepare the transfection assemblies (i.e., the addition/mixing method) have an impact on their physico-chemical features and this, in turn, affects their transfection effectiveness in vitro. Accordingly, any time a benchmark transfectant is used to gauge the effectiveness of another gene delivery vector, the materials used and the procedures followed should be disclosed with a suitable level of detail.

## 3. Experimental Strategies and In Vitro Transfection Assays

### 3.1. Cell Type and Culture Conditions

The vast majority of the transfection studies in vitro have been performed on adherent cell monolayers in multiwell culture plates. A large array of different replicating cells (i.e., immortalized cell lines, primary cells, and cancer cells), from different donor species (e.g., human, murine, monkey), and from diverse tissue types (e.g., endothelium, kidney, muscle) have been used for this purpose [68,137]. Because immortalized cell lines display high(er) proliferative rates, that is, short(er) doubling time, they are the first-choice option to check for the effectiveness of gene delivery systems. This is because the nuclear membrane temporarily disappears during each mitotic event in such a way that the pDNA becomes inherently accessible to the transcription machinery [78,138,139]. In this light, cell lines are easy-to-transfect cells because they multiply fast. Conversely, primary cells progressively take longer and longer to duplicate because they undergo senescence, and therefore are harder to transfect and require greater pDNA doses to reach even lower transfection efficiencies than cell lines [96]. Of note, pDNA-transfected cells transiently express the foreign gene; that is, it does not get integrated into the genome. As a result, no pDNA replication takes place over time. Consequently, the transgene is expressed only for a certain finite period of time and then is lost through cell division. Thus, on a per-cell basis, the shorter the doubling time, the greater the transfection efficiency and the less lasting the transgene expression.

Furthermore, cells require a certain confluence level to behave at best. Indeed, cell-to-cell interactions, in other words, direct cell-to-cell contact and communication, and secretion of diffusible factors between physically separated cells are a basic need for cell growth and division [140,141]. That is why the optimal cell density has to be properly selected to carry out transfection experiments, as pointed out in a recent paper [62]. 

Mycoplasma contamination is one of the major concerns that laboratories and commercial facilities employing cell lines have to face [142,143,144]. Indeed, mycoplasma are a common cause of cell contamination affecting about every fourth cell culture and endangering almost all aspects of cell physiology. Possible effects include the induction of chromosomal abnormalities, the disruption of DNA and RNA synthesis, changes in membrane antigenicity, the inhibition of cell proliferation and metabolism due to nutrient withdrawal, changes in gene expression profiles, and cell death, which result in decreased transfection rates [145,146]. These extensive effects of mycoplasma contamination on cultured cells make it clear that any data derived from mycoplasma-contaminated cell cultures, or any time cells are not verified mycoplasma-negative, are of questionable accuracy and should be treated with caution and suspicion. There are no tricks to avoiding mycoplasma contamination and other serious cell culture concerns. As cell culture techniques and applications become more complex, one must be aware of the impact of poorly controlled or suboptimal cell culture procedures. In this light, the doubling time, the passage number, and the cell confluence level are reliable indexes of how cells behave [147] and conceivably respond to transfection and, as such, they should be made explicit in the paper. Besides, in order to mitigate the risk of mycoplasma contamination, microbiological monitoring (e.g., through polymerase chain reaction (PCR)-based testing, [148,149,150]) is routinely required throughout cell culture and transfection. In this regard, the entire scientific community is rethinking mycoplasma contaminations, and high-impact factor journals either strongly recommend or even require regular cell testing. Obtaining and using low-passage cell lines from one of the world’s leading repositories is a sure way to work and publish with confidence.

### 3.2. Transfection Conditions

In vitro transfection experiments aim to assess the overall performance of non-viral gene delivery vectors. Transfection assays have evolved through the past decades and are nowadays performed following in-home protocols and rely on different read-out systems. All this makes it difficult to directly compare the results of one researcher with those of another.

The amount of DNA, and consequently of complexes, to be delivered to cells is a key experimental factor to be taken into account when looking for the best possible transfection outcomes. Although gene delivery experiments are usually carried out over a very narrow range of pDNA doses, the literature agrees on the existence of a causal relationship between the transfection efficiency and the dose of NAs utilized in transfection [137]. Recently, we and others have pinpointed the pDNA dose of 160–320 ng/cm^2^ as the most effective in different cell lines (HeLa, COS-7, and HepG2 cells) transfected with both PEI isomers [62,137]. 

**Figure 6:**
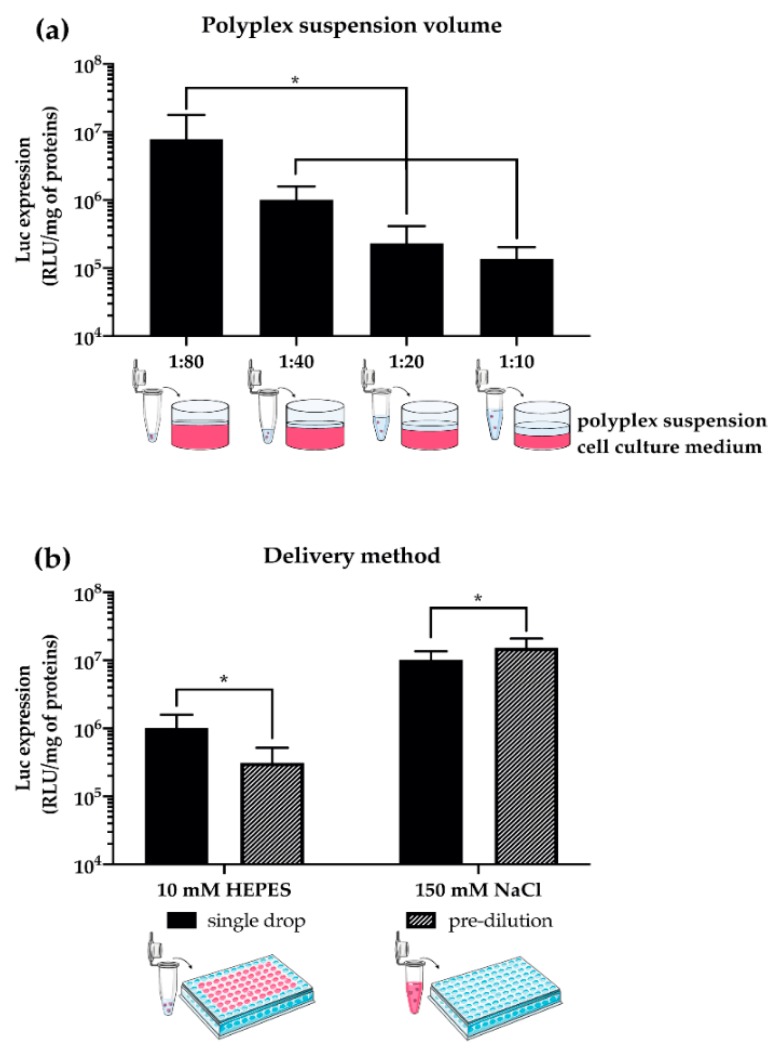
Effect of the volume of polyplex suspension and the delivery method on the transfection efficiency of pDNA/*l*PEI complexes prepared at N/P 30 in L929 cells. (**a**) Transfection efficiency of pDNA/*l*PEI complexes as a function of the polyplex volume:medium volume ratio. Complexes were prepared by mixing 160 ng/cm^2^ of pGL3 with *l*PEI solutions prepared in 10 mM Hepes in a final transfection volume of 1.28, 2.5, 5.12, and 10 μL, corresponding to 1:80, 1:40, 1:20, and 1:10 (*v/v*) ratios, respectively. The final volume of cell culture medium was 100 μL/well. (**b**) Transfection efficiency of pDNA/*l*PEI complexes as a function of the delivery method. Complexes were prepared by mixing 160 ng/cm^2^ of pGL3 with the *l*PEI in 10 mM Hepes in a final transfection volume of 2.56 μL/well and (i) directly added to culture medium in every well (i.e., single drop) or (ii) pre-diluted in the cell culture medium and next added to every well (i.e., pre-dilution). Results are expressed as mean ± SD (*n* ≥ 3) (* *p* < 0.05).

Furthermore, other parameters herein disclosed, such as the volume ratio between the polyplex suspension and the culture medium used during transfection of adherent cells (hereinafter referred to as the polyplex volume:medium volume ratio), and the way complexes are dispensed to cells in culture (Figure 6) have a striking effect on the transfection behavior of gene delivery vectors [62,63,108,113,125,128,151,152,153,154]. Specifically, the lower the transfectant volume:medium volume ratio (e.g., ≤1:40, which corresponds to deliver ≤2.5 µL of complexes to cells cultured in a 96-multiwell plate format with 100 µL/well of medium), the greater the transfection efficiency of polyplexes (Figure 6a). This is probably because cells are at their best, and thus are most permissive to transfection, when the concentration of the culture medium constituents is as close as possible to the standard culture conditions [137]. Besides, due to the presence of salts (i.e., ions) and proteins (i.e., polyions) in the cell culture medium, polyplexes may aggregate and become bigger right after their addition to the well. This results in greater transfection efficiencies because larger complexes settle and interact faster with cells [46,68,122].

Yet, the way polyplexes are added to cells may also dramatically affect the transfection efficiency. By and large, the delivery of complexes to cells is obtained through the addition of the transfection suspension to the cells cultured in the culture medium [62,96], or the culture vessel in which the cells were plated is emptied, polyplexes are first pre-diluted in the culture medium and then added to the cells [57,64]. As a general observation, *l*PEI-polyplexes prepared in 10 mM Hepes have smaller D_H_ and are more effective when directly added to the transfection well, while bigger particles (e.g., those prepared in 150 mM NaCl) benefit from the pre-dilution step in cell culture medium before they are incubated with cells (Figure 6b).

In overall terms, we herein point to the way complexes are prepared (e.g., the complexation buffer used) and the way polyplexes are delivered to cells as means to fine-tune the transfection effectiveness of a gene delivery vector. Because they are seldom considered as worthy, but their impact is huge, such details should be thoroughly described in any work on this matter.

### 3.3. Evaluation of Transfection Effectiveness: A Trade-off Between Transfection Efficiency and Cytotoxicity

One issue concerning the in vitro screening of transfectants pertains to the assays used to assess gene delivery efficacy. The effectiveness of non-viral vectors is causally linked to their ability to cross the cellular membrane and release the genetic cargo to allow transgene expression [46,70,155]. Quantitative and correlative measurements are therefore used to assess the safety and the efficacy of the delivery technologies [156,157,158].

Transfection efficiency is typically evaluated by analyzing the expression of a luminescent protein (i.e., firefly luciferase [62]), a fluorescent protein (i.e., GFP [159]), or other easily detectable proteins such as the β-galactosidase [137], or a combination thereof [160]. Depending on the transgene delivered, the read-out system allows direct detection of the cells expressing the protein of interest, as for fluorescent proteins, through flow cytometry (FCM) and imaging techniques, or indirectly [68,137]. The latter relies on collecting the chemiluminescence signal arising from the enzymatic conversion of a substrate into a product, as in the case of luciferase. Albeit these reporter genes are often used interchangeably in transfection experiments, each transgene and respective assay has a different sensitivity and its own metrics [161]. For instance, fluorescent proteins are good descriptors of the transfection efficiency at the single cells level, which is typically defined as the percentage of cells expressing the transgene-encoded protein [162,163,164]. Conversely, luciferase expression provides relevant information about pDNA expression levels within a cell population but not in single cells, as the chemiluminescence is expressed as arbitrary luminescence units per milligram of proteins in cell lysates [165,166,167,168]. Accordingly, each of these metrics is not, unfortunately, a comprehensive estimator of the efficiency of a given transfectant, if considered individually. In this regard, van Gaal and co-workers showed how different transgenes and read-out systems may reveal differences in the onset, level, and peak of expression [137].

Hence, it follows that the assessment of the transfection efficiency of a given transfectant by means of at least two different transgenes and read-out technologies allows a deeper and wider characterization of its gene delivery performance. 

The other side of the coin is the toxicity related to the gene delivery system. As a rule of thumb, the higher the transfection efficiency, the greater the cytotoxicity of a given gene delivery vector [169,170], and PEIs are not an exception. Contrary to popular belief, PEI is typically used in transfection experiments in vitro at concentrations far below the toxicity threshold, as depicted in Figures S1, S2, S4, and S5. 

The toxicity induced by non-viral gene delivery vectors and assemblies is generally assessed by means of colorimetric commercial test kits, such as Alamar Blue^®^, MTT, and XTT [117,152,153,171]. These cell viability assays make use of oxidation-reduction indicators that undergo a color change in response to the cellular metabolic reduction so that one can get quantitative information on the viability of transfected cells. Of note, gene delivery vectors are chemicals and, as such, they may interfere with colorimetric assays that thus provide faulty data [172,173,174]. Besides, lactate dehydrogenase (LDH), a method used to evaluate the cell toxicity by measuring the activity of cytoplasmic enzymes released by damaged cells, and live–dead staining coupled to FCM, are sometimes used as well.

Overall, a broad spectrum of cell viability/cytotoxicity assays is currently used in the field of non-viral gene delivery. The selection of the appropriate method among those available is important for obtaining accurate and reliable results. When selecting the cytotoxicity and cell viability assays to be used in the study, different parameters have to be considered, such as test compounds, detection mechanisms, specificity, and sensitivity.

## 4. Conclusions

The best way to compare the overall effectiveness in vitro of new gene delivery vectors between laboratories is to gauge their performances with respect to those of suitable benchmarks of seemingly well-known behavior. PEIs, together with commercially available reagents and kits such as those belonging to the Lipofectamine series and jetPEI^®^, have largely been used in a number of studies in this regard [57,60,69,175,176,177,178]. The way any benchmark transfectant behaves and performs is, however, critically dependent on a variety of parameters and in-home experimental conditions. Herein above, we sought to provide a catalog of relevant transfection conditions that were found optimal for the gold standard and internal reference, 25 kDa *l*PEI, and which can be profitably replicated across in vitro studies for the sake of comparison. Because there is no good science in bad models, it is our truthful hope that this review may provide fertile ground for the implementation of standardized protocols for an unbiased evaluation of the transfection effectiveness in vitro of more and more effective gene delivery reagents, and help scientists advance their work at a much faster pace.

## Figures and Tables

**Table 1:** The major types of NAs used in gene delivery applications and their specific features.

Nucleic Acid	Description	Site of Action	Applications/Pathway
pDNAs (also called chimeras) [1,9]	large circular dsDNAs (<10 kbp)	nucleus	nuclear localization followed by transgene expression under specific promoters to induce protein expression
mRNAs [1,9]	large ssRNAs (<10 kbp)	cytosol	positive regulation of protein expression
short regulatory RNAs (siRNAs/miRNAs/shRNA) [1,9]	short regulatory RNA (15–30 nt)	cytosol	RNA interference mechanisms to shorten mRNA half-life and downregulate translation
ASOs [10]	short DNA, RNA or analogs (15–30 nt)	cytosol and nucleus	RNA alteration to reduce, restore, or modify protein expression

dsDNA = double stranded DNA; bp = base pair; ssRNA = single stranded RNA; nt = nucleotide.

**Table 2:** Overview of the different transfection technologies for gene delivery applications.

Strategy		Description	Pros	Cons
Physical/mechanical methods [27,29,42]	electroporation	application of an electric field by voltage pulses to induce transient cell membrane poration	high efficiency; low costs; high reproducibility; ability to transfer large size DNA	tissue/cell damage; invasiveness; some DNA instability
	sonoporation	use of highly-focused ultrasounds to trigger transient cell membrane poration	non-invasiveness; possibility to be used in combination with microbubbles/non-viral vectors	low efficiency; low reproducibility; tissue/cell damage
	optoporation	use of short ultra-focused laser pulses to induce transient cell membrane poration	high efficiency; high spatial precision	tissue/cell damage; low irradiation area; poor penetration of the laser pulses
	magnetofection	application of a magnetic field to ease the transfer of NAs-coated paramagnetic particles into cells	high efficiency; non-invasiveness; possibility to be used in combination with non-viral vectors	poor efficiency with naked DNA; possible agglomeration of magnetic particles
	microinjection	direct injection of NAs into single cells by means of a needle	high efficiency; simplicity; reproducibility; low cytotoxicity; ability to transfer large size DNA	time consuming; inability to transfect large number of cells
	gene gun	propulsion of NAs-coated particles towards the target site	high efficiency; safety	tissue/cell damage; poor penetration of particles
Viral vectors [2,39,43,44]	adenoviruses (AdVs)	non-enveloped dsDNA–virus able to carry ≤8 kbp DNA	efficient in a broad range of host cells	high immunogenicity; transient expression
	adeno-associated viruses (AAVs)	non-enveloped recombinant ssDNA–virus with a small carrying capacity (≤4 kbp)	efficient in a broad range of host cells; non-inflammatory/pathogenic	small carrying capacity
	retroviruses	enveloped ssRNA-carrying virus with ≤8 kbp RNA capacity	long-term expression	limited tropism to dividing cells; random integration
	lentiviruses	enveloped ssRNA-carrying virus with ≤8 kbp RNA capacity	efficient in a broad range of host cells; long-term expression	potential oncogenic responses
	herpes simplex viruses (HSV)-1	enveloped dsDNA–virus with >30 kbp carrying capacity	large packing capacity; efficient in a broad range of host cells	potential inflammatory responses; transient expression
Non-viral vectors [11,45,46,47,48]	inorganic nanoparticles	metal-based nanoparticles of different size and shapes	possibility of functionalization; low cytotoxicity	instability; toxicity
	cation lipids	lipids able to self-assemble with NAs to give lipoplexes	tunable features; safety; low cytotoxicity	low transfection efficiency
	cationic polymers	polymers able to self-assemble with NAs to give polyplexes	tunable features; possibility of functionalization; mild cytotoxicity; stability in protein-rich media; low cytotoxicity	low transfection efficiency

## Supplementary Materials

The following are available online at https://www.mdpi.com/1999-4923/12/2/183/s1, S1. Experimental section; S2. Results; Figure S1: Cytotoxicity of complexes prepared at different N/P ratio; Figure S2: Cytotoxicity of pDNA/*l*PEI complexes prepared in different buffers; Figure S3: Effect of the complexation method on the physico-chemical characteristics of pDNA/*l*PEI complexes; Figure S4: Cytotoxicity of pDNA/*l*PEI complexes on L929 cells as a function of the complexation method; Figure S5: Cytotoxicity of pDNA/*l*PEI complexes on L929 cells as a function of the cell culture volume ratio and delivery method; Table S1: Physico-chemical characteristics of complexes prepared in different buffers; S3. References.
