# Supplementary material for: Non-Viral in Vitro Gene Delivery: It is Now Time to Set the Bar!
Source: Pharmaceutics. 2020 Feb 21;12(2):183. doi: 10.3390/pharmaceutics12020183 (PMC7076396; doi:10.3390/pharmaceutics12020183)
Supplement: Supplementary file 1 [file pharmaceutics-12-00183-s001.pdf]

*pharmaceutics*

*Supporting Information*

# Non-Viral in Vitro Gene Delivery: It is Now Time to Set the Bar!

**Nina Bono [1,†], Federica Ponti [1,2,†], Diego Mantovani [2] and Gabriele Candiani [1,*]**

[1] GenT Lab, Department of Chemistry, Materials and Chemical Engineering "G. Natta", Politecnico di Milano, 20131 Milan, Italy; nina.bono@polimi.it (N.B.); federica.ponti@polimi.it (F.P.)

[2] Laboratory for Biomaterials and Bioengineering, Canada Research Chair I in Biomaterials and Bioengineering for the Innovation in Surgery, Department of Min-Met-Materials Engineering & Research Center of CHU de Quebec, Division of Regenerative Medicine, Laval University, Quebec City, QC, Canada; diego.mantovani@gmn.ulaval.ca

† These authors equally contributed to this work

* Correspondence: gabriele.candiani@polimi.it; Tel.: +39-02-2399-3181

Academic Editor: Gabriele Candiani
Received: 3 February 2020; Accepted: date; Published: date

**Abstract:** Transfection by means of non-viral gene delivery vectors is the cornerstone of modern gene delivery. Despite the resources poured into the development of ever more effective transfectants, improvement is still slow and limited. Of note, the performance of any gene delivery vector in vitro is strictly dependent on several experimental conditions specific to each laboratory. The lack of standard tests has thus largely contributed to the flood of inconsistent data underpinning the reproducibility crisis. A way researchers seek to address this issue is by gauging the effectiveness of newly synthesized gene delivery vectors with respect to benchmarks of seemingly well-known behavior. However, the performance of such reference molecules is also affected by the testing conditions. This survey points to non-standardized transfection settings and limited information on variables deemed relevant in this context as the major cause of such misalignments. This review provides a catalog of conditions optimized for the gold standard and internal reference, 25 kDa polyethyleneimine, that can be profitably replicated across studies for the sake of comparison. Overall, we wish to pave the way for the implementation of standardized protocols in order to make the evaluation of the effectiveness of transfectants as unbiased as possible.

**Keywords:** non-viral gene delivery; cationic polymers; PEI; polyplexes; in vitro transfection; physico-chemical characterization; variability; reproducibility; standardization

**Table of contents**

1. Experimental section ..... 3
1.1. Materials ..... 3
1.2. Preparation of pDNA ..... 3
1.3. Preparation of PEI solutions ..... 3
1.4. Preparation of PEI/pDNA complexes ..... 3
1.5. Evaluation of PEI complexation ability ..... 4
1.6. Physico-chemical characterization of polyplexes ..... 4
1.7. In vitro transfection experiments ..... 4
1.7.1. Cell culture ..... 4
1.7.2. In vitro cell transfection ..... 4
1.7.3. Evaluation of cytotoxicity ..... 5
1.7.4. Evaluation of transfection efficiency ..... 5
1.8. Statistical analysis ..... 5
2. Results ..... 5
2.1. Figure S1: Cytotoxicity of complexes prepared at different N/Ps. ..... 5
2.2. Table S1: Physico-chemical characteristics of complexes prepared in different buffers. ..... 6
2.3. Figure S2: Cytotoxicity of pDNA/lPEI complexes prepared in different buffers. ..... 7
2.4. Figure S3: Effect of the complexation method on the physico-chemical characteristics of pDNA/lPEI complexes. ..... 8
2.5. Figure S4: Cytotoxicity of pDNA/lPEI complexes as a function of the complexation method. 9
2.6. Figure S5: Cytotoxicity of pDNA/lPEI complexes as a function of the polyplex volume:medium volume ratio and the delivery method. ..... 10
3. References ..... 11

## 1. Experimental section

### *1.1. Materials*

Plasmid DNA (pDNA) encoding for the modified firefly luciferase (pGL3-Control Vector, 5.2 kbp) and Luciferase Assay System were purchased from Promega (Milan, Italy), while plasmid purification kit was from Qiagen (Milan, Italy). L929 cell line (murine fibroblast from subcutaneous connective tissue; ATCC®-CCL-1) was from the American Type Tissue Culture Collection, while *Escherichia coli* (*E. coli*) DH5*α* (cat. nr. 9027) was from Takara Bio (Otsu, Japan). AlamarBlue Cell Viability Assay® was from Life Technologies Italia (Monza, Italy), while BCA Protein Assay Kit was from ThermoFisher (Monza, Italy). 25 kDa linear *l*PEI (cat. nr. 23966) was purchased from Polyscience (Eppelheim, Germany). All the other chemicals were from Merck Life Science S.r.l. (Rome, Italy), if not differently specified.

### *2.2. Preparation of pDNA*

*E. coli* were transformed with pGL3-Control Vector and amplified in Luria-Bertani (LB) broth (Life Technologies Italia, Monza, Italy) at 37 °C under shaking. The pDNA was isolated and purified using a Maxiprep Qiagen kit according to the supplier's guidelines. The plasmid was next diluted at a concentration of 250 ng/μL in 0.1x TE buffer (1 mM Tris, pH 8; 0.1 mM EDTA), then the concentration and purity were assessed by measuring the $OD_{260}/OD_{280}$ by means of a spectrophotometer (Nanodrop 2000c, Fisher Scientific, Illkirch, France). The pDNA solution was stored at -20°C until use.

### *2.3. Preparation of PEI solutions*

25 kDa *l*PEI solution was prepared in deionized water ($dH_2O$) at a concentration of 1 mg/mL, and the pH was adjusted to 7.0. Afterwards, the solution was diluted in Hepes buffer (final concentration of buffer: 10 mM) to give a stock solution of 0.86 mg of PEI/mL (corresponding to an amine concentration ([N]) of 20 mM). The stock solution was stored at 4°C until use.

### *2.4. Preparation of PEI/pDNA complexes*

Before complexation, *l*PEI and pDNA solutions were warmed to room temperature (r.t.).

Complexes were prepared combining the pDNA and PEI solutions at the desired polymer concentration to yield different N/Ps (i.e., 1, 2, 3, 5, 10, 20, 30, 40 and 60), followed by a 20 min-incubation at r.t.. N/P is defined as the amine moles (N, cationic moiety) of the polymers with respect to the phosphate moles (P, anionic moiety) of a given quantity of DNA.

Polyplex suspensions were prepared in in 10 mM Hepes (pH 7.0), 10 mM Hepes supplemented with 5% (w/v) of glucose (hereafter referred to as HBG, pH 7.0), 150 mM NaCl (pH 7.4), and deionized water ($dH_2O$).

To assess the influence of the complexation method, polyplexes were prepared in 10 mM Hepes *i)* by adding the pDNA solution to the cationic polymer (CP) solution at the stoichiometric ratio of 1:10 (v/v), *ii)* by adding the PEI solution to the pDNA solution at the 10:1 (v/v) ratio, and *iii)* by adding pre-diluted pDNA solution to the PEI solution at the stoichiometric ratio of 1:1 (v/v).

Polyplexes were also invariably prepared adding the pDNA to the PEI at the stoichiometric ratio of 1:10 (v/v) by *i)* rigorous pipetting for 10 s, *ii)* by single dripping and *iii)* by vortexing the two for 10 s.

Just after their preparation, complexes underwent physico-chemical characterization and were used for *in vitro* transfection assays.

### 2.5. Evaluation of PEI complexation ability

The ability of *l*PEI to bind and complex pDNA was evaluated by means of the fluorescence-exclusion titration assay, as described elsewhere [1]. Briefly, polyplexes were invariably prepared by mixing 0.25 µg of pGL3 in 1.0 µL of 200× SYBR Green I ($\lambda_{ex}$ = 497 nm, $\lambda_{em}$ = 520 nm) with 11.8 µL of *l*PEI solutions at different concentrations, yielding a final DNA concentration of 20 ng/µL and different N/P ratios. Afterward, polyplexes were incubated for 20 min at r.t., then diluted 1:16 (v/v) in 10 mM Hepes. Fluorescence measurements (n = 3 per condition) were performed with a Synergy H1 spectrophotometer (BioTek, Italy) in 384-multiwell black plates. Data are expressed as relative fluorescence normalized to the fluorescence of uncomplexed pDNA, as follows:

$$F(\%) = \frac{F_{sample} - F_{blank}}{F_{DNA\ only} - F_{blank}} \times 100 \quad (1)$$

### 2.6. Physico-chemical characterization of polyplexes

For physico-chemical characterization, 50 µL of pDNA/*l*PEI complexes were prepared as described herein above. Afterwards, polyplexes were diluted 1:10 in the specific buffer and allowed to equilibrate for 5 min before measurements. The size (expressed as hydrodynamic diameter, $D_H$) and surface charge (expressed as zeta potential, $z_P$) of polyplexes were measured at 25°C by Dynamic Light Scattering (DLS) and Electrophoretic Light Scattering (ELS), respectively, using a Malvern Zetasizer Nano instrument (Malvern, Italy), fitted with a 5 mV HeNe laser ($\lambda$ = 633 nm) and a scattering angle of 173°.

### 2.7. In vitro transfection experiments

#### 2.7.1. Cell culture

L929 cells were expanded in T75 flasks in Dulbecco's Modified Eagle's Medium (DMEM) supplemented with 1 mM sodium pyruvate, 10 mM Hepes buffer, 100 U/mL penicillin, 0.1 mg/mL streptomycin, 2 mM glutamine and 10% (v/v) fetal bovine serum (FBS) (hereafter referred to as complete DMEM, cDMEM) in a humidified atmosphere and 5% $CO_2$ at 37 °C (hereafter referred to as standard culture conditions). L929 cells were regularly checked for mycoplasma contamination according to a procedure reported in [2].

#### 2.7.2. In vitro cell transfection

For any transfection experiment, L929 cells were kept between passage 5 and 10. The cells were seeded onto 96-well plates at a density of 2 ′ $10^4$ cells/cm$^2$ and cultured in standard culture conditions. Twenty-four hrs after seeding, 160 ng/cm$^2$ of pGL3 were complexed with *l*PEI as described herein above.

To assess the influence of the volume ratio between the polyplex suspension and the culture medium on the transfection efficiency, the cells were challenged with different volumes of polyplex suspensions, i.e., 1.28 mL (corresponding to a 1:80 (v/v) ratio in 98.72 mL/well of cell culture medium to give a final volume of 100 mL/well in a 96-multiwell plate format), 2.5 mL (corresponding to a 1:40 (v/v) ratio in 97.5 mL/well of cell culture medium), 5.12 mL (corresponding to a 1:20 (v/v) ratio in 94.88 mL/well of cell culture medium), and 10 mL (corresponding to a 1:10 v/v ratio in 90 mL/well of cell culture medium).

To assess the influence of the delivery method on the transfection efficiency, complexes were delivered to cells i) by adding the polyplexes (2.5 µL/well in a 96-multiwell plate format) to each cell culture well pre-filled with fresh cDMEM (97.5 µL/well) and ii) by pre-diluting the complexes in cDMEM (50 µL/well) before the addition to the cell culture wells pre-filled with fresh cDMEM (50 µL/well).

2.7.3. Evaluation of cytotoxicity

Twenty-four hrs-post transfection, the cytotoxicity was evaluated by means of the Alamar Blue assay, according to the manufacturer's instructions. Briefly, the cDMEM was removed from each well that was loaded with 100 mL/well of 1′ resazurin dye solution in cDMEM. Next, plates were incubated in standard culture conditions for 2 hrs in the dark, then the fluorescence was read by means of a Synergy H1 spectrophotometer (BioTek, Italy) ($\lambda_{ex}$ = 540 nm; $\lambda_{em}$ = 595 nm). The viability of non-transfected cells (CTRL) was assigned to as 100% and cytotoxicity was determined according to the following equation:

$$\text{Cytotoxicity [\%]} = 100\% - \text{Viability [\%]} \quad (2)$$

2.7.4. Evaluation of transfection efficiency

The transgene expression was evaluated measuring the luciferase activity using the Luciferase Assay system, following the manufacturer's instructions. Briefly, the cells were washed with PBS and lysed with 110 mL/well of Cell Culture Lysis Reagent (Promega, Italy). After three freeze-thaw cycles to promote cell disruption, 20 mL of cell lysates were mixed with 50 mL of luciferase assay reagent, and the luminescence was measured by means of the chemiluminescence reader. The luminescence signal (Relative Light Units, RLU) was normalized to the total protein content of each sample, as determined by the BCA assay. Transfection efficiency was finally expressed as RLU/mg of protein.

*2.8. Statistical analysis*

Statistical analysis was carried out with GraphPad version 8 (GraphPad software, La Jolla, CA, USA). Data were initially analyzed using D'Agostino & Pearson omnibus normality test. Comparisons between groups were performed with one-way analysis of variance (ANOVA) and multiple t-tests. Significance was retained when $p < 0.05$. Data are expressed as mean ± standard deviation (SD). Experiments were performed at least in triplicate.

## 3. Results

*3.1. Figure S1: Cytotoxicity of complexes prepared at different N/Ps.*

Cytotoxicity of pDNA/*l*PEI complexes in L929 cells as a function of the N/P. The corresponding CP concentration is displayed in the top x-axis. Complexes were invariably prepared by adding 160 ng/cm² of pGL3 to *l*PEI in 10 mM Hepes. The cytotoxicity is expressed with respect to CTRL cells. Results are expressed as mean ± SD (n ³ 3).

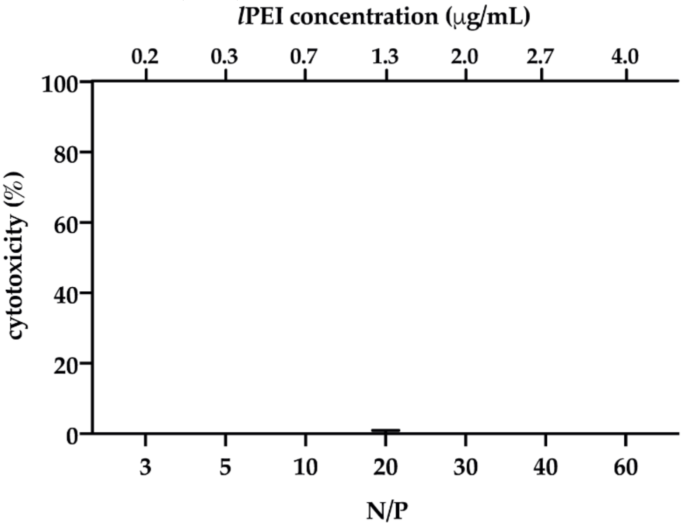

*3.2. Table S1: Physico-chemical characteristics of complexes prepared in different buffers.*

Hydrodynamic diameter ($D_H$) and zeta potential ($z_P$) of the polyplexes measured by Dynamic Light Scattering (DLS) and Electrophoretic Light Scattering (ELS), respectively. Polyplexes were invariably prepared at N/P 30 in 150 mM NaCl, 10 mM Hepes, HGB, and $dH_2O$ by adding the pDNA solution to the PEI solution (160 ng of pGL3/$cm^2$ in a 96-multiwell format), and mixed to PEI by pipetting. Measurements (n=3 per condition) were performed after a 5 min-equilibration step in each buffer.

| **polymer** | **150 mM NaCl** | | **10 mM Hepes** | | **HGB** | | **$dH_2O$** | |
|---|---|---|---|---|---|---|---|---|
| | **$D_H$ (nm)** | **$z_P$ (mV)** | **$D_H$ (nm)** | **$z_P$ (mV)** | **$D_H$ (nm)** | **$z_P$ (mV)** | **$D_H$ (nm)** | **$z_P$ (mV)** |
| 25 kDa *l*PEI | 875±106 | 25±4 | 154±22 | 19±3 | 142±30 | 22±3 | 159±26 | 26±3 |

*3.3. Figure S2: Cytotoxicity of pDNA/lPEI complexes prepared in different buffers.*

Cytotoxicity of pDNA/*l*PEI complexes in L929 cells, prepared in different buffers. Complexes were invariably prepared at N/P 30 by adding 160 ng/cm$^2$ of pGL3 to *l*PEI in different buffers. The cytotoxicity is expressed with respect to CTRL cells. Results are expressed as mean ± SD (n ³ 3).

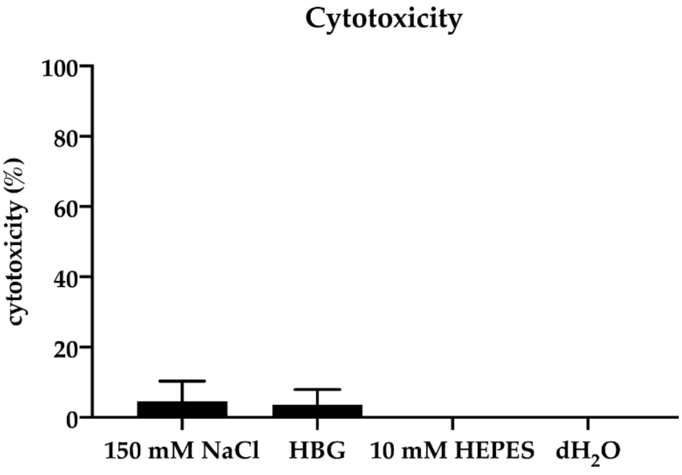

*3.4. Figure S3: Effect of the complexation method on the physico-chemical characteristics of pDNA/lPEI complexes.*

(a) Hydrodynamic diameter ($D_H$) and (b) zeta potential ($z_P$) of pDNA/*l*PEI complexes as a function of the order of mixing and volumes of *l*PEI and DNA solutions. Complexes at N/P 30 were prepared by mixing 1 mg of pDNA with the *l*PEI solution in 10 mM Hepes (DNA to PEI), or *vice versa* (PEI to DNA), then mixing the two solutions by rigorous pipetting, or by mixing equivolumes of DNA and PEI solutions (v/v). (c) $D_H$ and (d) $z_P$ of pDNA/*l*PEI complexes as a function of the complexation method. Complexes at N/P 30 were prepared by adding 1 mg of pDNA to *l*PEI in 10 mM Hepes by single dripping, rigorous pipetting (i.e., mixing), and vortexing. Measurements were carried out by means of a Dynamic Light Scattering (DLS) and Electrophoretic Light Scattering (ELS) apparatus. Results are expressed as mean ± SD (n ³ 3) (*p < 0.05).

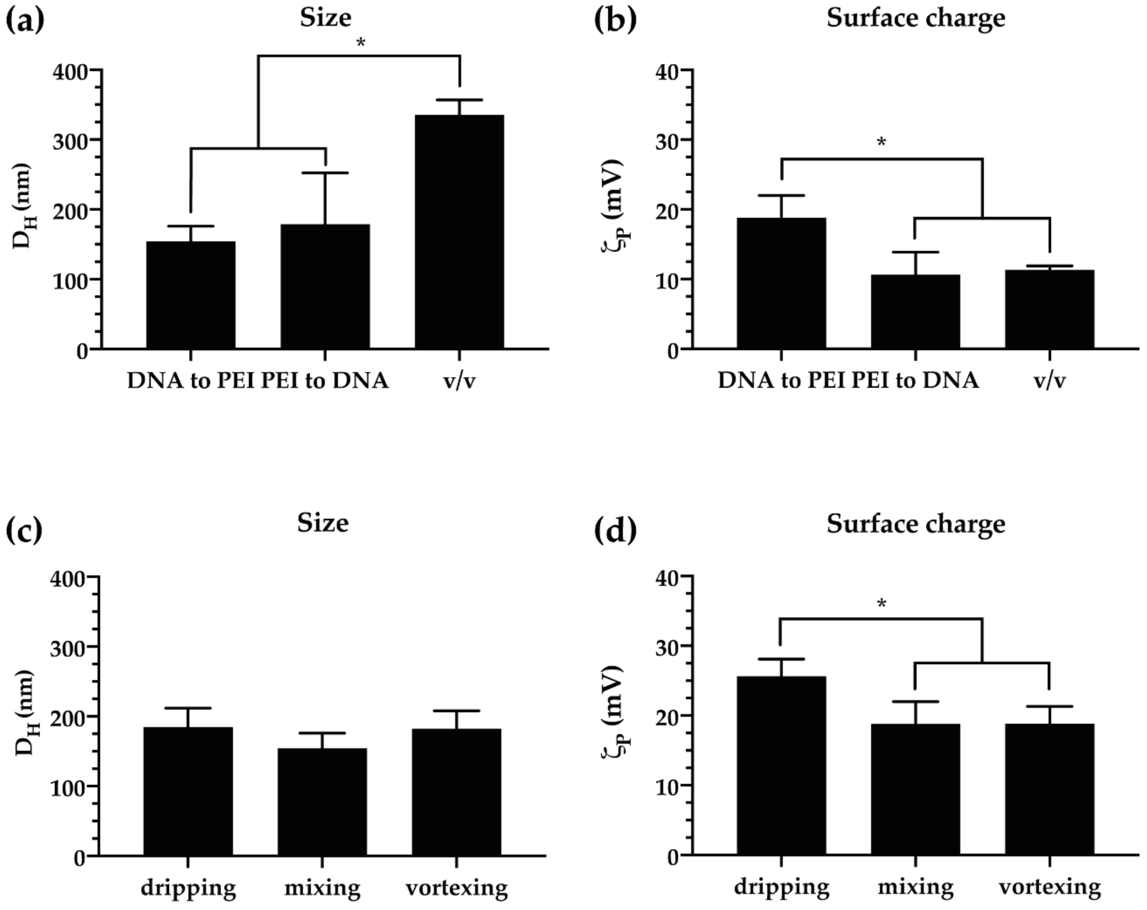

*3.5. Figure S4: Cytotoxicity of pDNA/lPEI complexes as a function of the complexation method.*

Cytotoxicity of pDNA/*l*PEI complexes in L929 cells as a function of (a) the order of addition of reagents and volumes of pDNA and *l*PEI solutions and (b) the complexation method. Complexes were prepared by adding 160 ng/cm$^2$ of pGL3 to *l*PEI (DNA to PEI) or *vice versa* (PEI to DNA) or by mixing equivolumes of DNA and PEI solutions (v/v) (a), and by single dripping, rigorous pipetting or vortexing (b). Cytotoxicity is expressed with respect to CTRL cells. Results are expressed as mean ± SD (n $^{3}$ 3).

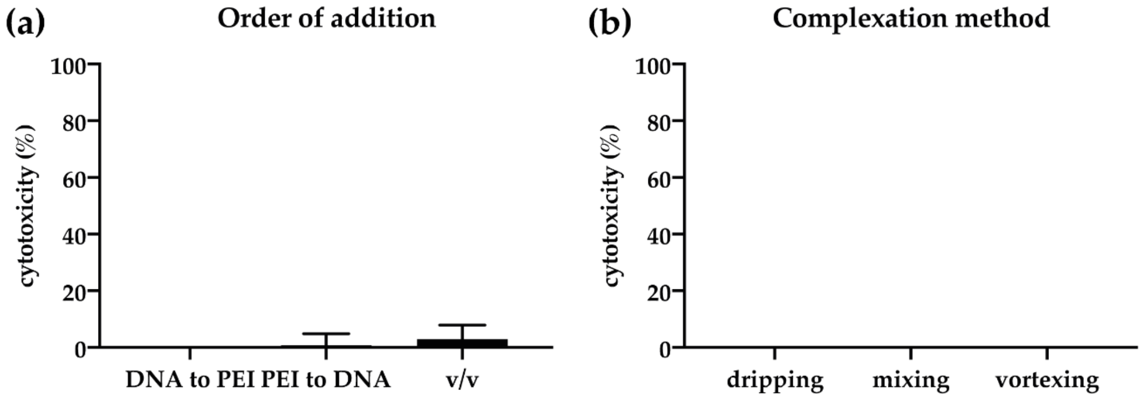

*3.6. Figure S5: Cytotoxicity of pDNA/lPEI complexes as a function of the polyplex volume:medium volume ratio and the delivery method.*

Cytotoxicity of pDNA/*l*PEI complexes in L929 cells as a function of (a) the polyplex suspension:cell culture volume ratio and (b) the delivery method. (a) Complexes at N/P 30 were prepared by mixing 160 ng/cm$^2$ of pGL3 with *l*PEI solutions prepared in 10 mM Hepes in a final transfection volume of 1.28, 2.5, 5.12, and 10 mL, corresponding to 1:80, 1:40, 1:20, and 1:10 (v/v) polyplex suspension to culture volume ratio, respectively. (b) Complexes at N/P 30 were prepared by adding 160 ng/cm$^2$ of pGL3 to the *l*PEI in 10 mM Hepes in a final transfection volume of 2.56 mL/well and i) directly added to culture medium in every well (i.e., single drop) or ii) pre-diluted in the cell culture medium and delivered to each well (i.e., pre-dilution). Cytotoxicity is expressed with respect to CTRL cells. Results are expressed as mean ± SD (n ³ 3).

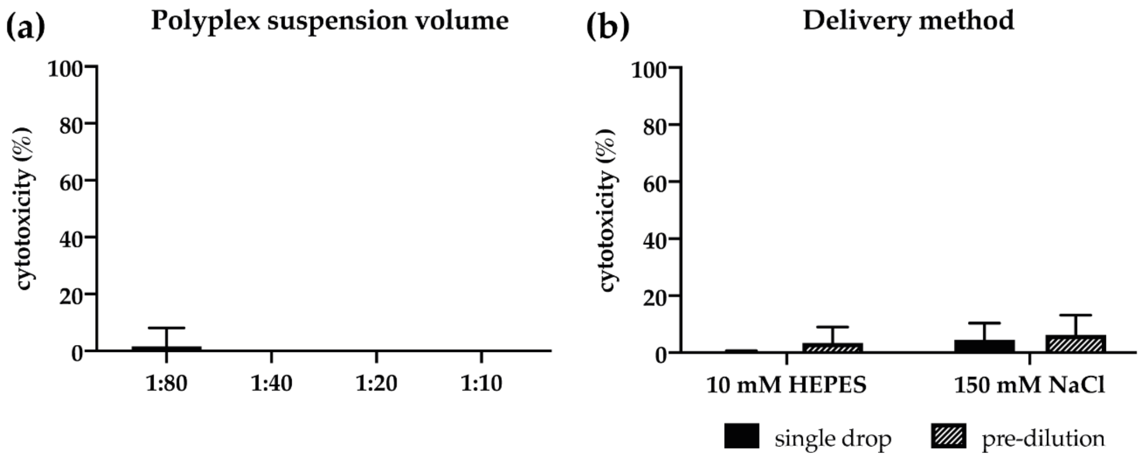

**References**


1. Bono, N.; Pennetta, C.; Bellucci, M.C.; Sganappa, A.; Malloggi, C.; Tedeschi, G.; Candiani, G.; Volonterio, A. Role of Generation on Successful DNA Delivery of PAMAM-(Guanidino)Neomycin Conjugates. *ACS Omega* **2019**, *4*, 6796–6807.
2. Tang, J.; Hu, M.; Lee, S.; Roblin, R. A polymerase chain reaction based method for detecting Mycoplasma/Acholeplasma contaminants in cell culture. *J. Microbiol. Methods* **2000**, *39*, 121–126.


© 2020 by the authors. Licensee MDPI, Basel, Switzerland. This article is an open access article distributed under the terms and conditions of the Creative Commons Attribution (CC BY) license (http://creativecommons.org/licenses/by/4.0/).
